# Cuckoo Search with Lévy Flights for Weighted Bayesian Energy Functional Optimization in Global-Support Curve Data Fitting

**DOI:** 10.1155/2014/138760

**Published:** 2014-05-28

**Authors:** Akemi Gálvez, Andrés Iglesias, Luis Cabellos

**Affiliations:** ^1^Department of Applied Mathematics and Computational Sciences, E.T.S.I. Caminos, Canales y Puertos, University of Cantabria, Avenida de los Castros s/n, 39005 Santander, Spain; ^2^Department of Information Science, Faculty of Sciences, Toho University, 2-2-1 Miyama, Funabashi 274-8510, Japan; ^3^Institute of Physics of Cantabria (IFCA), Avenida de los Castros s/n, 39005 Santander, Spain

## Abstract

The problem of data fitting is very important in many theoretical and applied fields. In this paper, we consider the problem of optimizing a weighted Bayesian energy functional for data fitting by using global-support approximating curves. By global-support curves we mean curves expressed as a linear combination of basis functions whose support is the whole domain of the problem, as opposed to other common approaches in CAD/CAM and computer graphics driven by piecewise functions (such as B-splines and NURBS) that provide local control of the shape of the curve. Our method applies a powerful nature-inspired metaheuristic algorithm called *cuckoo search*, introduced recently to solve optimization problems. A major advantage of this method is its simplicity: cuckoo search requires only two parameters, many fewer than other metaheuristic approaches, so the parameter tuning becomes a very simple task. The paper shows that this new approach can be successfully used to solve our optimization problem. To check the performance of our approach, it has been applied to five illustrative examples of different types, including open and closed 2D and 3D curves that exhibit challenging features, such as cusps and self-intersections. Our results show that the method performs pretty well, being able to solve our minimization problem in an astonishingly straightforward way.

## 1. Introduction

The problem of data fitting is very important in many theoretical and applied fields [1–4]. For instance, in computer design and manufacturing (CAD/CAM), data points are usually obtained from real measurements of an existing geometric entity, as it typically happens in the construction of car bodies, ship hulls, airplane fuselage, and other free-form objects [5–15]. This problem also appears in the shoes industry, archeology (reconstruction of archeological assets), medicine (computed tomography), computer graphics and animation, and many other fields. In all these cases, the primary goal is to convert the real data from a physical object into a fully usable digital model, a process commonly called* reverse engineering*. This allows significant savings in terms of storage capacity and processing and manufacturing time. Furthermore, the digital models are easier and cheaper to modify than their real counterparts and are usually available anytime and anywhere.

Depending on the nature of these data points, two different approaches can be employed:* interpolation* and* approximation*. In the former, a parametric curve or surface is constrained to pass through all input data points. This approach is typically employed for sets of data points that come from smooth shapes and that are sufficiently accurate. On the contrary, approximation does not require the fitting curve or surface to pass through all input data points, but just close to them, according to some prescribed distance criteria. The approximation scheme is particularly well suited for the cases of highly irregular sampling and when data points are not exact, but subjected to measurement errors. In real-world problems the data points are usually acquired through laser scanning and other digitizing devices and are, therefore, subjected to some measurement noise, irregular sampling, and other artifacts [12, 13]. Consequently, a good fitting of data should be generally based on approximation schemes rather than on interpolation [16–20].

There are two key components for a good approximation of data points with curves: a proper choice of the approximating function and a suitable parameter tuning. Due to their good mathematical properties regarding evaluation, continuity, and differentiability (among many others), the use of polynomial functions (especially splines) is a classical choice for the approximation function [16, 17, 23–27]. In general, the approximating curves can be classified as global-support and local-support. By global-support curves we mean curves expressed as a linear combination of basis functions whose support is the whole domain of the problem. As a consequence, these curves exhibit a global control, in the sense that any modification of the shape of the curve in a particular location is propagated throughout the whole curve. This is in clear contrast to the local-support approaches that have become prevalent in CAD/CAM and computer graphics, usually driven by piecewise functions (such as B-splines and NURBS) that provide local control of the shape of the curve [23, 28]. In this work we are particularly interested to explore the performance of the global-support approach by using different global-support basis functions for our approximating curves.

### 1.1. Main Contributions and Structure of the Paper

In this paper, we consider the problem of optimizing a weighted Bayesian energy functional for data fitting by using global-support approximating curves. In particular, our goal is to obtain the global-support approximating curve that fits the data points better while keeping the number of free parameters of the model as low as possible. To this aim, we formulate this problem as a minimization problem by using a weighted Bayesian energy functional for global-support curves. This is one of the major contributions of this paper. Our functional is comprised of two competing terms aimed at minimizing the fitting error between the original and the reconstructed data points while simultaneously minimizing the degrees of freedom of the problem. Furthermore, the functional can be modified and extended to include various additional constraints, such as the fairness and smoothness constraints typically required in many industrial operations in computer-aided manufacturing, such as CNC (computer numerically controlled) milling, drilling, and machining [4, 5, 12].

Unfortunately, our formulation in previous paragraph leads to a nonlinear continuous optimization problem that cannot be properly addressed by conventional mathematical optimization techniques. To overcome this limitation, in this paper we apply a powerful nature-inspired metaheuristic algorithm called cuckoo search, introduced in 2009 by Yang and Deb to solve optimization problems [21]. The algorithm is inspired by the obligate interspecific brood parasitism of some cuckoo species that lay their eggs in the nests of host birds of other species. Since its inception, the cuckoo search (specially its variant that uses Lévy flights) has been successfully applied in several papers reported recently in the literature to difficult optimization problems from different domains. However, to the best of our knowledge, the method has never been used so far in the context of geometric modeling and data fitting. This is also one of the major contributions of this paper.

A critical problem when using metaheuristic approaches concerns the parameter tuning, which is well known to be time-consuming and problem-dependent. In this regard, a major advantage of the cuckoo search with Lévy flights is its simplicity: it only requires two parameters, many fewer than other metaheuristic approaches, so the parameter tuning becomes a very simple task. The paper shows that this new approach can be successfully applied to solve our optimization problem. To check the performance of our approach, it has been applied to five illustrative examples of different types, including open and closed 2D and 3D curves that exhibit challenging features, such as cusps and self-intersections. Our results show that the method performs pretty well, being able to solve our minimization problem in an astonishingly straightforward way.

The structure of this paper is as follows: in Section 2 some previous work in the field is briefly reported. Then, Section 3 introduces the basic concepts and definitions along with the description of the problem to be solved. The fundamentals and main features of the cuckoo search algorithm are discussed in Section 4. The proposed method for the optimization of our weighted Bayesian energy functional for data fitting with global-support curves is explained in Section 5. Some other issues such as the parameter tuning and some implementation details are also reported in that section. As the reader will see, the method requires a minimal number of control parameters. As a consequence, it is very simple to understand, easy to implement and can be applied to a broad variety of global-support basis functions. To check the performance of our approach, it has been applied to five illustrative examples for the cases of open and closed 2D and 3D curves exhibiting challenging features, such as cusps and self-intersections, as described in Section 6. The paper closes with the main conclusions of this contribution and our plans for future work in the field.

## 2. Previous Works

The problem of curve data fitting has been the subject of research for many years. First approaches in the field were mostly based on numerical procedures [1, 29, 30]. More recent approaches in this line use error bounds [31], curvature-based squared distance minimization [26], or dominant points [18]. A very interesting approach to this problem consists in exploiting minimization of the energy of the curve [32–36]. This leads to different functionals expressing the conditions of the problem, such as fairness, smoothness, and mixed conditions [37–40]. Generally, research in this area is based on the use of nonlinear optimization techniques that minimize an energy functional (often based on the variation of curvature and other high-order geometric constraints). Then, the problem is formulated as a multivariate nonlinear optimization problem in which the desired form will be the one that satisfies various geometric constraints while minimizing (or maximizing) a measure of form quality. A variation of this formulation consists in optimizing an energy functional while simultaneously minimizing the number of free parameters of the problem and satisfying some additional constraints on the underlying model function. This is the approach we follow in this paper.

Unfortunately, the optimization problems given by those energy functionals and their constraints are very difficult and cannot be generally solved by conventional mathematical optimization techniques. On the other hand, some interesting research carried out during the last two decades has shown that the application of artificial intelligence techniques can achieve remarkable results regarding such optimization problems [6, 8, 10, 11, 14]. Most of these methods rely on some kind of neural networks, such as standard neural networks [8] and Kohonen's SOM (self-organizing maps) nets [10]. In some other cases, the neural network approach is combined with partial differential equations [41] or other approaches [42]. The generalization of these methods to functional networks is also analyzed in [6, 11, 14]. Other approaches are based on the application of nature-inspired metaheuristic techniques, which have been intensively applied to solve difficult optimization problems that cannot be tackled through traditional optimization algorithms. Examples include artificial immune systems [43], bacterial foraging [44], honey bee algorithm [45], artificial bee colony [46], firefly algorithm [47, 48], and bat algorithm [49, 50]. A previous paper in [51] describes the application of genetic algorithms and functional networks yielding pretty good results. Genetic algorithms have also been applied to this problem in both the discrete version [52, 53] and the continuous version [7, 54]. Other metaheuristic approaches applied to this problem include the use of the popular particle swarm optimization technique [9, 24], artificial immune systems [55, 56], firefly algorithm [57, 58], estimation of distribution algorithms [59], memetic algorithms [60], and hybrid techniques [61].

## 3. Mathematical Preliminaries

In this paper we assume that the solution to our fitting problem is given by a model function Φ(*ξ*) defined on a finite interval domain [*ν*_1_, *ν*_2_]. Note that in this paper vectors are denoted in bold. We also assume that Φ(*ξ*) can be mathematically represented as a linear combination of the so-called blending functions:

(1) $$\begin{array}{c} \Phi \left(\xi \right)=\overset{\delta }{\underset{\alpha =1}{\sum }}\Theta_{\alpha }\psi_{\alpha }\left(\xi \right),\;\xi \in \left[\nu_{1},\nu_{2}\right]. \end{array}$$

In this work, the family of blending functions {*ψ*_*α*_(*ξ*)}_*α*_ in (1) is assumed to be linearly independent and to form a basis of the vector space of functions of degree ≤*δ* − 1 on [*ν*_1_, *ν*_2_]. In this paper we consider the case in which all functions {*ψ*_*α*_(*ξ*)}_*α*_ have their support on the whole domain [*ν*_1_, *ν*_2_]. Without loss of generality, we can also assume that [*ν*_1_, *ν*_2_] is the unit interval [0,1]. In practical terms, this means that the blending functions provide a global control of the shape of the approximating curve (these functions are usually referred to as global-support functions), as opposed to the alternative case of local control given by the piecewise representation that is characteristic of popular curves such as B-splines and NURBS. Typical examples of global-support basis functions are

1. the canonical polynomial basis: *ψ*_*α*_(*ξ*) = *ξ* ^*α*−1^;
2. the Bernstein basis: $\psi_{\alpha }(\xi )=\left(\begin{array}{c} \delta -1\\ \alpha -1 \end{array}\right)\xi^{\alpha -1}{\left(1-\xi \right)}^{\delta -\alpha }$.

Other examples include the Hermite polynomial basis, the trigonometric basis, the hyperbolic basis, the radial basis, and the polyharmonic basis.

Let us suppose now that we are given a finite set of data points {Δ_*β*_}_*β*=1,…,*ζ*_ in a *D*-dimensional space (usually *D* = 2 or *D* = 3). Our goal is to obtain a global-support approximating curve that best fits these data points while keeping the number of degrees of freedom as low as possible. This leads to a difficult minimization problem involving two different (and competing) factors: the fitting error at the data points and the number of free parameters of the model function. In this paper, we consider the RMSE (root mean square error) as the fitting error criterion. The number of free parameters is computed by following a Bayesian approach (see [62] for further details). This is a very effective procedure to penalize fitting models with too many parameters, thus preventing data overfitting [63]. Therefore, our optimization problem consists in minimizing the following weighted Bayesian energy functional:

(2) L=ζ2log⁡(∑β=1ζΩβ[Δβ−∑α=1δΘαψα(ρβ)]2)+ζ·γ2(2δ−12)log⁡(ζ),

where we need a parameter value *ρ*_*β*_ to be associated with each data point Δ_*β*_. Equation (2) is comprised of two terms: the first one computes the fitting error to the data points, while the second one plays the role of a penalty term in order to reduce the degrees of freedom of the model. The penalty term also includes a real positive multiplicative factor *γ* used to modulate how much this term will affect the whole energy functional.

This functional *L* can be modified or expanded to include any additional constrain in our model. For instance, it is very common in many engineering domains such as computer-aided ship-hull design, car-body styling, and turbine-blade design to request conditions such as fairness or smoothness. In our approach, these conditions can readily be imposed by adding different energy functionals adapted to the particular needs. Suppose that instead of reducing the degrees of freedom of our problem, the smoothness of the fitting curve is required. This condition is simply incorporated to our model by replacing the penalty term in (2) by the strain energy functional as follows:

(3) L=ζ2log⁡(∑β=1ζΩβ[Δβ−∑α=1δΘαψα(ρβ)]2)+ζ·γ2(λ∫||Φ′′(ξ)||2dξ).

Considering the vectors **Ξ**_*α*_ = (*ψ*_*α*_(*ρ*_1_),…, *ψ*_*α*_(*ρ*_*ζ*_))^*T*^, with *α* = 1,…, *δ*, where (·)^*T*^ means transposition, **Ξ** = (**Ξ**_1_,…, **Ξ**_*δ*_), Δ = (Δ_1_,…, Δ_*ζ*_), **Ω** = (**Ω**_1_,…, **Ω**_*ζ*_), and Θ = (Θ_1_,…, Θ_*δ*_)^*T*^, (2) can be written in matricial form as

(4) L=Ω·ΔT·Δ−Ω·ΘT·ΞT·Δ−Ω·ΔT·Ξ·Θ+Ω·ΘT·ΞT·Ξ·Θ.

Minimization of *L* requires differentiating (4) with respect to Θ and equating to zero to satisfy the first-order conditions, leading to the following system of equations (called the* normal equations*):

(5) $$\begin{array}{c} \Xi^{T}\cdot \Xi \cdot \Theta =\Xi^{T}\cdot \Delta . \end{array}$$

In general, the blending functions {*ψ*_*α*_(*ξ*)}_*α*_ are nonlinear in *ξ*, leading to a strongly nonlinear optimization problem, with a high number of unknowns for large sets of data points, a case that happens very often in practice. Our strategy for solving the problem consists in applying the cuckoo search method to determine suitable parameter values for the minimization of functional *L* according to (2). The process is performed iteratively for a given number of iterations. Such a number is another parameter of the method that has to be calculated in order to run the algorithm until the convergence of the minimization of the error is achieved.

## 4. The Cuckoo Search Algorithm

Cuckoo search (CS) is a nature-inspired population-based metaheuristic algorithm originally proposed by Yang and Deb in 2009 to solve optimization problems [21]. The algorithm is inspired by the obligate interspecific brood parasitism of some cuckoo species that lay their eggs in the nests of host birds of other species with the aim of escaping from the parental investment in raising their offspring. This strategy is also useful to minimize the risk of egg loss to other species, as the cuckoos can distribute their eggs amongst a number of different nests. Of course, sometimes it happens that the host birds discover the alien eggs in their nests. In such cases, the host bird can take different responsive actions varying from throwing such eggs away to simply leaving the nest and build a new one elsewhere. However, the brood parasites have at their turn developed sophisticated strategies (such as shorter egg incubation periods, rapid nestling growth, and egg coloration or pattern mimicking their hosts) to ensure that the host birds will care for the nestlings of their parasites.

This interesting and surprising breeding behavioral pattern is the metaphor of the cuckoo search metaheuristic approach for solving optimization problems. In the cuckoo search algorithm, the eggs in the nest are interpreted as a pool of candidate solutions of an optimization problem, while the cuckoo egg represents a new coming solution. The ultimate goal of the method is to use these new (and potentially better) solutions associated with the parasitic cuckoo eggs to replace the current solution associated with the eggs in the nest. This replacement, carried out iteratively, will eventually lead to a very good solution of the problem.

In addition to this representation scheme, the CS algorithm is also based on three idealized rules [21, 22].

1. Each cuckoo lays one egg at a time and dumps it in a randomly chosen nest.
2. The best nests with high quality of eggs (solutions) will be carried over to the next generations.
3. The number of available host nests is fixed, and a host can discover an alien egg with a probability *p*_*a*_ ∈ [0,1]. In this case, the host bird can either throw the egg away or abandon the nest so as to build a completely new nest in a new location.

For simplicity, the third assumption can be approximated by a fraction *p*_*a*_ of the *n* nests being replaced by new nests (with new random solutions at new locations). For a maximization problem, the quality or fitness of a solution can simply be proportional to the objective function. However, other (more sophisticated) expressions for the fitness function can also be defined.

Based on these three rules, the basic steps of the CS algorithm can be summarized as shown in the pseudocode reported in Algorithm 1. Basically, the CS algorithm starts with an initial population of *n* host nests and it is performed iteratively. In the original proposal, the initial values of the *j*th component of the *i*th nest are determined by the expression *x*_*i*_^*j*^(0) = rand · (up_*i*_^*j*^ − low_*i*_^*j*^) + low_*i*_^*j*^, where up_*i*_^*j*^ and low_*i*_^*j*^ represent the upper and lower bounds of that *j*th component, respectively, and rand represents a standard uniform random number on the open interval (0,1). Note that this choice ensures that the initial values of the variables are within the search space domain. These boundary conditions are also controlled in each iteration step.

For each iteration *g*, a cuckoo egg *i* is selected randomly and new solutions **x**_*i*_(*g* + 1) are generated by using the Lévy flight, a kind of random walk in which the steps are defined in terms of the step lengths, which have a certain probability distribution, with the directions of the steps being isotropic and random. According to the original creators of the method, the strategy of using Lévy flights is preferred over other simple random walks because it leads to better overall performance of the CS. The general equation for the Lévy flight is given by

(6) $$\begin{array}{c} x_{i}\left(g+1\right)=x_{i}\left(g\right)+\alpha ⊕\text{levy}\left(\lambda \right), \end{array}$$

where *g* indicates the number of the current generation and *α* > 0 indicates the step size, which should be related to the scale of the particular problem under study. The symbol ⊕ is used in (6) to indicate the entrywise multiplication. Note that (6) is essentially a Markov chain, since next location at generation *g* + 1 only depends on the current location at generation *g* and a transition probability, given by the first and second terms of (6), respectively. This transition probability is modulated by the Lévy distribution as

(7) $$\begin{array}{c} \text{levy}\left(\lambda \right)∼g^{-\lambda },\;\left(1<\lambda \leq 3\right), \end{array}$$

which has an infinite variance with an infinite mean. Here the steps essentially form a random walk process with a power-law step-length distribution with a heavy tail. From the computational standpoint, the generation of random numbers with Lévy flights is comprised of two steps: firstly, a random direction according to a uniform distribution is chosen; then, the generation of steps following the chosen Lévy distribution is carried out. The authors suggested using the so-called Mantegna's algorithm for symmetric distributions, where “symmetric” means that both positive and negative steps are considered (see [64] for details). Their approach computes the factor

(8) $$\begin{array}{c} \hat{\phi }={\left(\frac{\Gamma \left(1+\hat{\beta }\right)\cdot \sin\left(\left(\pi \cdot \hat{\beta }\right)/2\right)}{\Gamma \left(\left(\left(1+\hat{\beta }\right)/2\right)\cdot \hat{\beta }\cdot 2^{\left(\hat{\beta }-1\right)/2}\right)}\right)}^{1/\hat{\beta }}, \end{array}$$

where Γ denotes the Gamma function and $\hat{\beta }=3/2$ in the original implementation by Yang and Deb [22]. This factor is used in Mantegna's algorithm to compute the step length *s* as

(9) $$\begin{array}{c} \varsigma =\frac{u}{{\left|v\right|}^{1/\hat{\beta }}}, \end{array}$$

where *u* and *v* follow the normal distribution of zero mean and deviation *σ*_*u*_^2^ and *σ*_*v*_^2^, respectively, where *σ*_*u*_ obeys the Lévy distribution given by (8) and *σ*_*v*_ = 1. Then, the stepsize *η* is computed as

(10) $$\begin{array}{c} \eta =0.01\varsigma \left(x-x_{\text{best}}\right), \end{array}$$

where *ς* is computed according to (9). Finally, **x** is modified as **x** ← **x** + *η* · *Υ*, where *Υ* is a random vector of the dimension of the solution **x** and that follows the normal distribution *N*(0,1).

The CS method then evaluates the fitness of the new solution and compares it with the current one. In case the new solution brings better fitness, it replaces the current one. On the other hand, a fraction of the worse nests (according to the fitness) are abandoned and replaced by new solutions so as to increase the exploration of the search space looking for more promising solutions. The rate of replacement is given by the probability *p*_*a*_, a parameter of the model that has to be tuned for better performance. Moreover, for each iteration step, all current solutions are ranked according to their fitness and the best solution reached so far is stored as the vector **x**_best_ (used, e.g., in (10)).

This algorithm is applied in an iterative fashion until a stopping criterion is met. Common terminating criteria are that a solution is found that satisfies a lower threshold value, that a fixed number of generations have been reached, or that successive iterations no longer produce better results.

## 5. The Method

We have applied the cuckoo search algorithm discussed in previous section to our optimization problem described in Section 3. The problem consists in minimizing the weighted Bayesian energy functional given by (2) for a given family of global-support blending functions. To this aim, we firstly need a suitable representation of the variables of the problem. We consider an initial population of *n* nests, representing the potential solutions of the problem. Each solution consists of a real-valued vector of dimension *D* · *δ* + 3*ζ* + 2 containing the parameters *ρ*_*β*_, vector coefficients Θ_*α*_, and weights *Ω*_*β*_, *δ*, and *γ*. The structure of this vector is also highly constrained. On one hand, the set of parameters {*ρ*_*β*_}_*β*_ is constrained to lie within the unit interval [0,1]. In computational terms, this means that different controls are to be set up in order to check for this condition to hold. On the other hand, the ordered structure of data points means that those parameters must also be sorted. Finally, weights are assumed to be strictly positive real numbers.

Regarding the fitness function, it is given by either the weighted Bayesian energy functional in (2) or by the weighted strain energy functional in (3), where the former penalizes any unnecessarily large number of free parameters for the model, while the latter imposes additional constraints regarding the smoothness of the fitting curve. Note also that the strength of the functionals can be modulated by the parameter *γ* to satisfy additional constraints.

### 5.1. Parameter Tuning

A critical issue when working with metaheuristic approaches concerns the choice of suitable parameter values for the method. This issue is of paramount importance since the proper choice of such values will largely determine the good performance of the method. Unfortunately, it is also a very difficult task. On one hand, the field still lacks sufficient theoretical results to answer this question on a general basis. On the other hand, the choice of parameter values is strongly problem-dependent, meaning that good parameter values for a particular problem might be completely useless (even counterproductive) for any other problem. These facts explain why the choice of adequate parameter values is so troublesome and very often a bottleneck in the development and application of metaheuristic techniques.

The previous limitations have been traditionally overcome by following different strategies. Perhaps the most common one is to obtain good parameter values empirically. In this approach, several runs or executions of the method are carried out for different parameter values and a statistical analysis is performed to derive the values leading to the best performance. However, this approach is very time-consuming, especially when different parameters influence each other. This problem is aggravated when the metaheuristic depends on many different parameters, leading to an exponential growth in the number of executions. The cuckoo search method is particularly adequate in this regard because of its simplicity. In contrast to other methods that typically require a large number of parameters, the CS only requires two parameters, namely, the population size *n* and the probability *p*_*a*_. This makes the parameter tuning much easier for CS than for other metaheuristic approaches.

Some previous works have addressed the issue of parameter tuning for CS. They showed that the method is relatively robust to the variation of parameters. For instance, authors in [21] tried different values for *n* = 5,10,15,20,50,100,150,250, and 500 and *p*_*a*_ = 0,0.01,0.05,0.1,0.15,0.2,0.25,0.4, and 0.5. They obtained that the convergence rate of the method is not very sensitive to the parameters used, implying that no fine adjustment is needed for the method to perform well. Our experimental results are in good agreement with these empirical observations. We performed several trials for the parameter values indicated above and found that our results do not differ significantly in any case. We noticed, however, that some parameter values are more adequate in terms of the number of iterations required to reach convergence. In this paper, we set the parameters *n* and *p*_*a*_ to 100 and 0.25, respectively.

### 5.2. Implementation Issues

Regarding the implementation, all computations in this paper have been performed on a 2.6 GHz Intel Core i7 processor with 8 GB RAM. The source code has been implemented by the authors in the native programming language of the popular scientific program MATLAB, version 2012a. We remark that an implementation of the CS method has been described in [21]. Similarly, a vectorized implementation of CS in MATLAB is freely available in [65]. Our implementation is strongly based (although not exactly identical) on that efficient open-source version of the CS.

## 6. Experimental Results

We have applied the CS method described in previous sections to different examples of curve data fitting. To keep the paper in manageable size, in this section we describe only five of them, corresponding to different families of global-support basis functions and also to open and closed 2D and 3D curves. In order to replicate the conditions of real-world applications, we assume that our data are irregularly sampled and subjected to noise. Consequently, we consider a nonuniform sampling of data in all our examples. Data points are also perturbed by an additive Gaussian white noise of small intensity given by a SNR (signal-to-noise ratio) of 60 in all reported examples.

**Figure 1:**
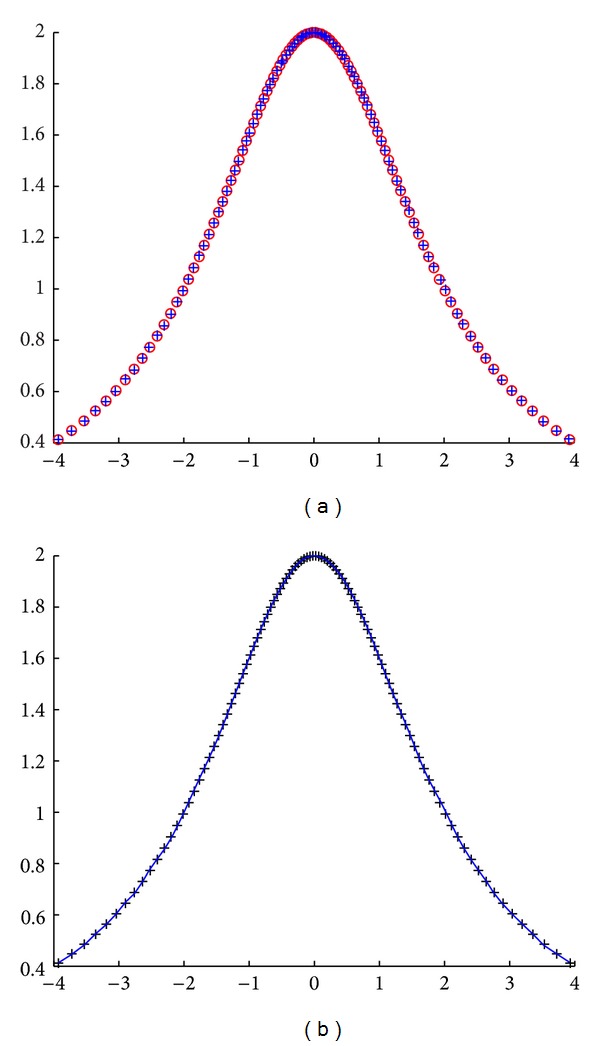
Application of the cuckoo search algorithm to the Agnesi curve: (a) original data points (red emptied circles) and reconstructed points (in blue plus symbol); (b) data points (black plus symbol) and fitting curve (solid blue line).

First example corresponds to a set of 100 noisy data points obtained by nonuniform sampling from the Agnesi curve. The curve is obtained by drawing a line *OB* from the origin through the circle of radius *r* and center (0, *r*) and then picking the point with the *y* coordinate of the intersection with the circle and the *x* coordinate of the intersection of the extension of line *OB* with the line *y* = 2*r*. Then, they are fitted by using the Bernstein basis functions. Our results are depicted in Figure 1(a), where the original data points are displayed as red emptied circles, whereas the reconstructed points appear as blue plus symbols. Note the good matching between the original and the reconstructed data points. In fact, we got a fitness value of 1.98646 × 10^−3^, indicating that the reconstructed curve fits the noisy data points with high accuracy. The average CPU time for this example is 3.01563 seconds. We also computed the absolute mean value of the difference between the original and the reconstructed data points for each coordinate and obtained good results: (9.569738 × 10^−4^, 1.776091 × 10^−3^). This good performance is also reflected in Figure 1(b), where the original data points and the reconstructed Bézier fitting curve are displayed as black plus symbols and a blue solid line, respectively.

**Figure 2:**
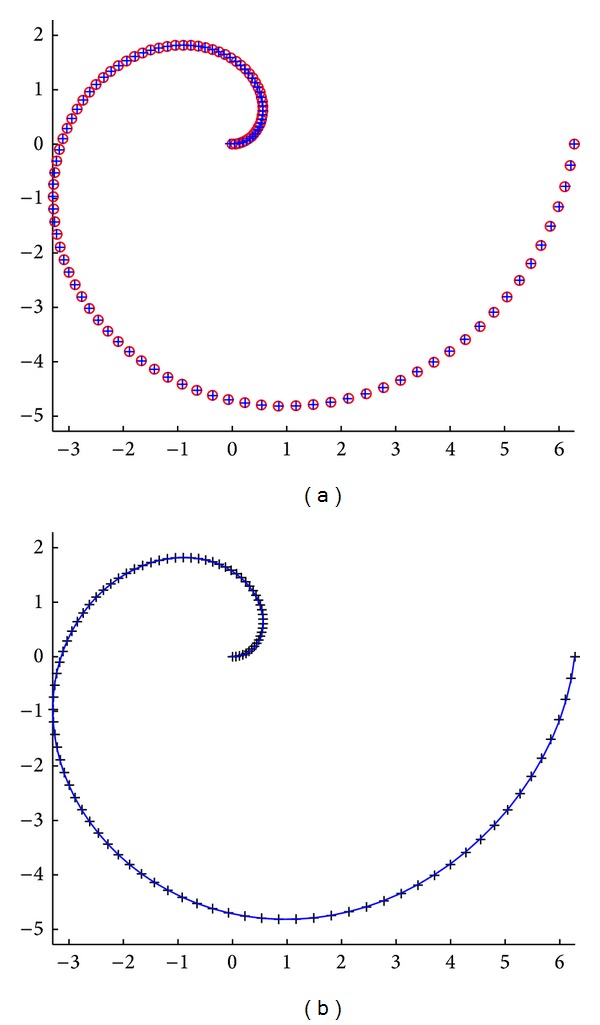
Application of the cuckoo search algorithm to the Archimedean spiral curve: (a) original data points (red emptied circles) and reconstructed points (in blue plus symbol); (b) data points (black plus symbol) and fitting curve (solid blue line).

Second example corresponds to the Archimedean spiral curve (also known as the arithmetic spiral curve). This curve is the locus of points corresponding to the locations over time of a point moving away from a fixed point with a constant speed along a line which rotates with constant angular velocity. In this example, we consider a set of 100 noisy data points from such a curve that are subsequently fitted by using the canonical polynomial basis functions. Our results for this example are depicted in Figure 2. We omit the interpretation of this figure because it is similar to the previous one. Once again, note the good matching between the original and the reconstructed data points. In this case we obtained a fitness value of 1.12398 × 10^−2^ for these data points, while the absolute mean value of the difference between the original and the reconstructed data points for each coordinate is (1.137795 × 10^−2^, 6.429596 × 10^−3^). The average CPU time for this example is 4.68752 seconds. We conclude that the CS method is able to obtain a global-support curve that fits the data points pretty well.

**Figure 3:**
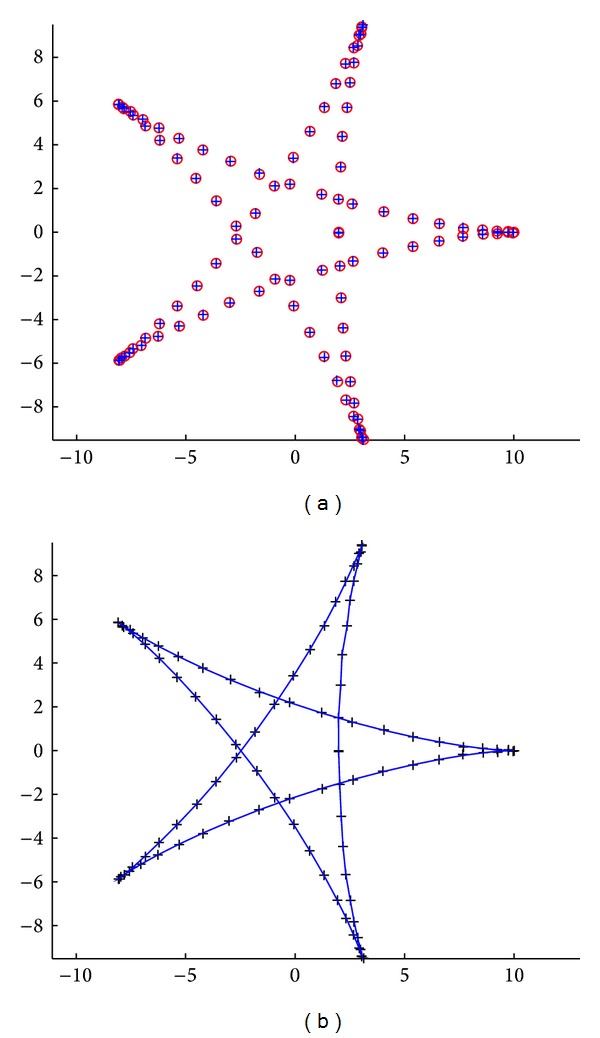
Application of the cuckoo search algorithm to the hypocycloid curve example: (a) original data points (red emptied circles) and reconstructed points (in blue plus symbol); (b) data points (black plus symbol) and fitting curve (solid blue line).

Third example corresponds to a hypocycloid curve. This curve belongs to a set of a much larger family of curves called the roulettes. Roughly speaking, a roulette is a curve generated by tracing the path of a point attached to a curve that is rolling upon another fixed curve without slippage. In principle, they can be any two curves. The particular case of a hypocycloid corresponds to a roulette traced by a point attached to a circle of radius *r* rolling around the inside of a fixed circle of radius *R*, where it is assumed that *R* = *k* · *r*. If *k* = *R*/*r* is a rational number, then the curve eventually closes on itself and has *R* cusps (i.e., sharp corners, where the curve is not differentiable). In this example, we consider a set of 100 noisy data points from the hypocycloid curve with 5 cusps. They are subsequently fitted by using the Bernstein basis functions.  Figure 3 shows our results graphically. In this case, the best fitness value is 2.00706 × 10^−3^, while the absolute mean value of the difference between the original and the reconstructed data points for each coordinate is (1.661867 × 10^−3^, 1.521872 × 10^−3^). The average CPU time for this example is 9.82813 seconds. In this case, the complex geometry of the curve, involving several cusps and self-intersections, leads to this relatively large CPU time in comparison with the previous (much simpler) examples. In fact, this example is very illustrative about the ability of the method to perform well even in case of nonsmooth self-intersecting curves.

**Figure 4:**
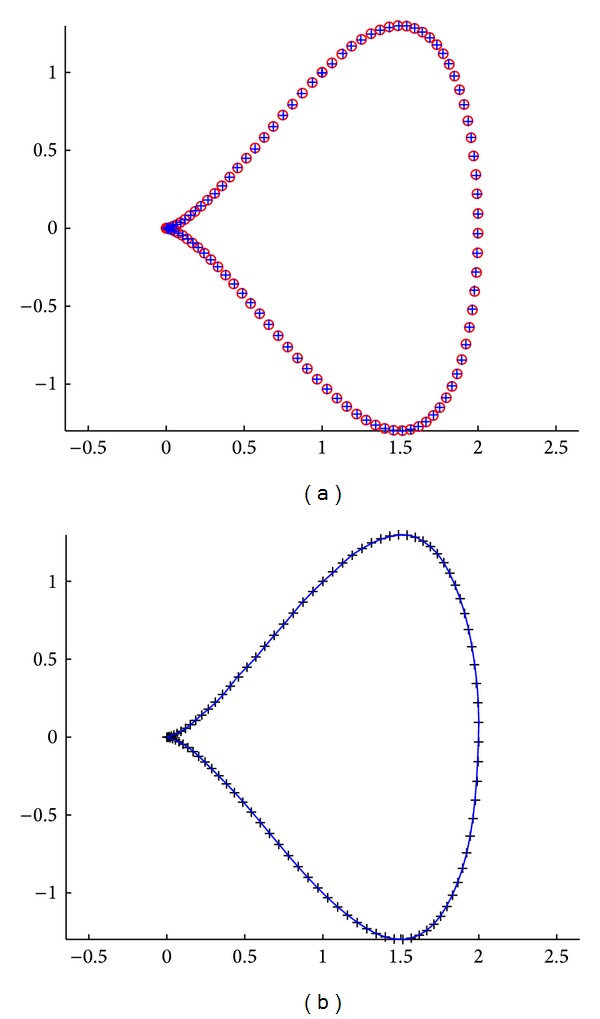
Application of the cuckoo search algorithm to the piriform curve example: (a) original data points (red emptied circles) and reconstructed points (in blue plus symbol); (b) data points (black plus symbol) and fitting curve (solid blue line).

Fourth example corresponds to the so-called piriform curve, which can be defined procedurally in a rather complex way. Once again, we consider a set of 100 noisy data points fitted by using the Bernstein basis functions. Our results are shown in Figure 4. The best fitness value in this case is 1.17915 × 10^−3^, while the absolute mean value of the difference between the original and the reconstructed data points for each coordinate is (8.64616 × 10^−4^, 5.873391 × 10^−4^). The average CPU time for this example is 3.276563 seconds. Note that this curve has a cusp in the leftmost part; moreover, the data points tend to concentrate around the cusp, meaning that the data parameterization is far from uniform. However, the method is still able to recover the shape of the curve with great detail.

**Figure 5:**
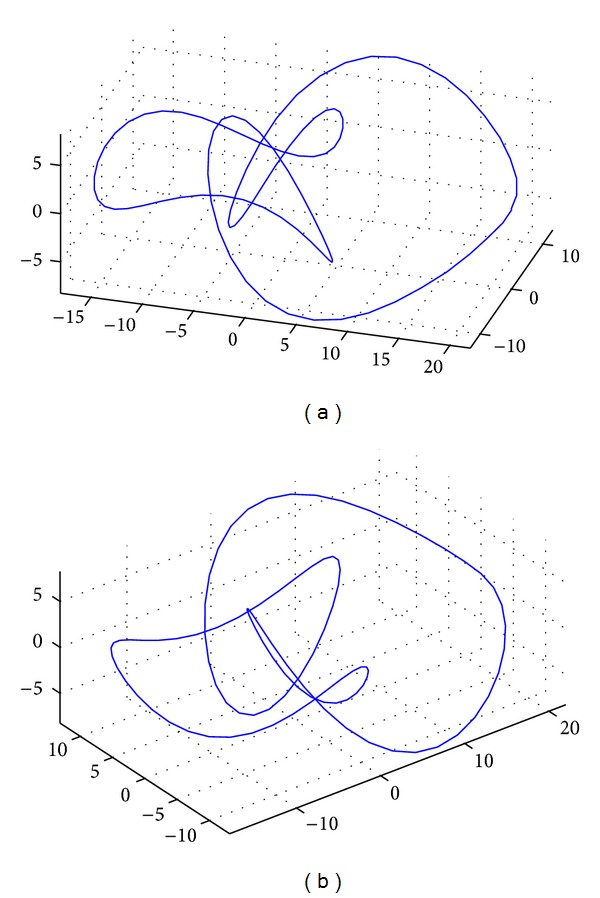
Two different viewpoints of the 3D Eight Knot curve.

**Figure 6:**
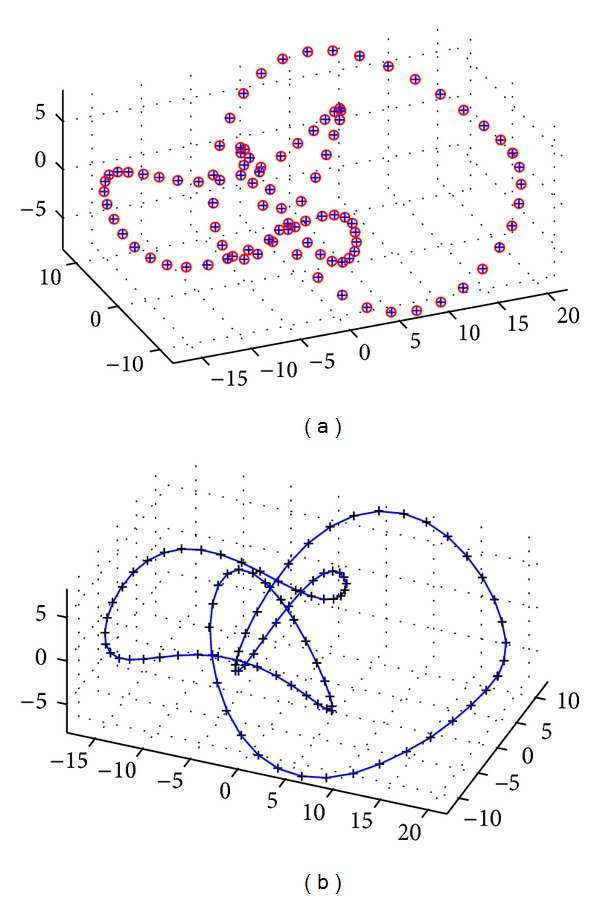
Application of the cuckoo search algorithm to the 3D Eight Knot curve example: (a) original data points (red emptied circles) and reconstructed points (in blue plus symbol); (b) data points (black plus symbol) and fitting curve (solid blue line).

The last example corresponds to a 3D closed curve called Eight Knot curve. Two images of the curve from different viewpoints are shown in Figure 5. The CS method is applied to a set of 100 noisy data points for the Bernstein basis functions. Our results are shown in Figure 6. The best fitness value in this case is 3.193634 × 10^−2^, while the absolute mean value of the difference between the original and the reconstructed data points for each coordinate is (2.7699870 × 10^−2^, 2.863125 × 10^−2^, 1.3710703 × 10^−2^). The average CPU time for this example is 8.75938 seconds.

## 7. Conclusions and Future Work

This paper addresses the problem of approximating a set of data points by using global-support curves while simultaneously minimizing the degrees of freedom of the model function and satisfying other additional constraints. This problem is formulated in terms of a weighted Bayesian energy functional that encapsulates all these constraints into a single mathematical expression. In this way, the original problem is converted into a continuous nonlinear multivariate optimization problem, which is solved by using a metaheuristic approach. Our method is based on the cuckoo search, a powerful nature-inspired metaheuristic algorithm introduced recently to solve optimization problems. Cuckoo search (especially its variant that uses Lévy flights) has been successfully applied to difficult optimization problems in different fields. However, to the best of our knowledge, this is the first paper applying the cuckoo search methodology in the context of geometric modeling and data fitting.

Our approach based on the cuckoo search method has been tested on five illustrative examples of different types, including open and closed 2D and 3D curves. Some examples also exhibit challenging features, such as cusps and self-intersections. They have been fitted by using two different families of global-support functions (Bernstein basis functions and the canonical polynomial basis) with satisfactory results in all cases. The experimental results show that the method performs pretty well, being able to solve our difficult minimization problem in an astonishingly straightforward way. We conclude that this new approach can be successfully applied to solve our optimization problem.

A major advantage of this method is its simplicity: cuckoo search requires only two parameters, many fewer than other metaheuristic approaches, so the parameter tuning becomes a very simple task. This simplicity is also reflected in the CPU runtime of our examples. Even though we are dealing with a constrained continuous multivariate nonlinear optimization problem and with curves exhibiting challenging features such as cusps and self-intersections, a typical single execution takes less than 10 seconds of CPU time for all the examples reported in this paper. In addition, the method is simple to understand, easy to implement and does not require any further pre-/postprocessing.

In spite of these encouraging results, further research is still needed to determine the advantages and limitations of the present method at full extent. On the other hand, some modifications of the original cuckoo search have been claimed to outperform the initial method on some benchmarks. Our implementation has been designed according to the specifications of the original method and we did not test any of its subsequent modifications yet. We are currently interested in exploring these issues as part of our future work. The hybridization of this approach with other competitive methods for better performance is also part of our future work.

## Figures and Tables

**Algorithm 1:**
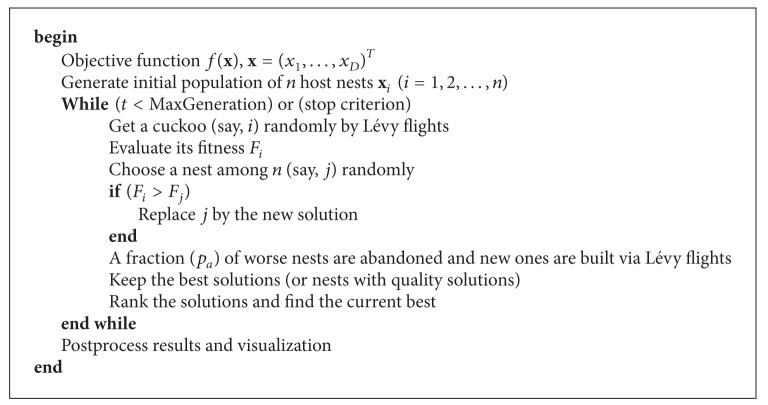
Cuckoo search algorithm via Lévy flights as originally proposed by Yang and Deb in [21, 22].
